# Associations between female sex-specific risk factors and cardiometabolic multimorbidity: evidence from the UK Biobank cohort study

**DOI:** 10.1186/s12889-026-28116-3

**Published:** 2026-06-12

**Authors:** Li Mao, Lijin Lin, Fang Lei, Yu Tian, Guoying Wang, Jinxing He, Juan Wan, Hui Liu

**Affiliations:** 1 Gannan Innovation and Translational Medicine Research Institute, State Key Laboratory of New Drug Discovery and Development for Major Diseases, Gannan Medical University, Building 4, Phase II of National High-level Talents Science and Technology Innovation Park, Zhanggong High-tech Zone, Ganzhou, Jiangxi 341000 China; 2 Medical Science Research Centre, Zhongnan Hospital of Wuhan University, Wuhan, Hubei China

**Keywords:** Cardiometabolic multimorbidity, Female, Menopause, Pregnancy

## Abstract

**Background:**

Hormonal changes throughout life in women are associated with menarche, pregnancy, lactation, and menopause. Cardiometabolic multimorbidity (CMM) has become increasingly common with population aging. However, the impact of such fluctuations on CMM has not been evaluated. This study aims to investigate the associations of female sex-specific risk factors with CMM and four cardiometabolic diseases.

**Methods:**

A total of 248,623 women from the UK Biobank without pre-existing cardiometabolic disease were included. Cox proportional hazard models were used to evaluate the association between female-specific risk factors and the incidence of CMM and the four cardiometabolic diseases. The UpSet plots were used to visualize combinations of female-specific risk factors, and then to evaluate the association between the combinations and accumulations of female risk factors and CMM.

**Results:**

The average age of participants was 56.4 years, with a median follow-up period of 13.6 years. After adjusting for multiple variables, early menarche was associated with an 11% higher risk for CMM incidence (HR, 1.11; 95% CI, 1.06–1.15). Early menopause (age < 35 years: HR, 1.80; 95% CI, 1.58–2.06; age 35–44 years: HR, 1.24; 95% CI, 1.17–1.32; age 45–49 years: HR, 1.09; 95% CI, 1.04–1.15), younger maternal age at first (age < 21 years: HR, 1.35; 95% CI, 1.28–1.42; age 21–25 years: HR, 1.13; 95% CI, 1.08–1.18) and last (age < 26 years: HR, 1.12; 95% CI, 1.06–1.18) live birth were associated with increased CMM risk, respectively. Women with three or more children had a higher risk of developing CMM (3 children: HR, 1.05; 95% CI, 1.00–1.10; ≥ 4 children HR, 1.19; 95% CI, 1.13–1.27). Additionally, a history of two or more stillbirths (HR, 1.30; 95% CI, 1.07–1.59) or spontaneous miscarriages (HR, 1.24; 95% CI, 1.15–1.34) was associated with elevated CMM risk, with similar findings for each cardiometabolic disease. Combinations or accumulations of sex-specific risk factors are associated with an increased risk of CMM.

**Conclusions:**

In summary, female sex-specific factors significantly influence the incidence of both CMM and each cardiometabolic disease.

## Introduction

With the growing proportion of aging individuals worldwide, multimorbidity has emerged as a critical public health issue, as it is linked to increased disability, mortality, reduced quality of life, and a higher disease burden [1–5]. Cardiometabolic multimorbidity (CMM), defined as the coexistence of at least two of the specified cardiometabolic diseases (type 2 diabetes, ischemic heart disease, stroke, and hypertension), represents one of the most prevalent and severe forms of multimorbidity [6]. Evidence suggests that at the age of 60 years, men with any two of cardiometabolic conditions experience an average reduction in life expectancy of 12 years, whereas women face an average reduction of 13 years [1]. Identifying and detecting risk factors for CMDs early is crucial for improving our understanding of these conditions, facilitating the early identification of high-risk individuals, and supporting the development of effective prevention strategies [7].

The complex relationship between reproductive health and the development of CMDs in women is gaining increasingly attention. Throughout a woman's life-from menarche to pregnancy and through menopause-significant fluctuations in sex hormones occur, impacting various aspects of cardiovascular health, including glucose and lipid metabolisms, cardiac energy metabolism and function, body composition, vascular function, and inflammatory responses [8–13]. An analysis involving 144,306 Finnish women demonstrated that those with adverse pregnancy outcomes had a heightened risk of stroke [14]. Other studies have indicated that certain sex-specific risk factors might be associated with an increased risk of type 2 diabetes in women [15, 16]. While the association between reproductive factors and cardiovascular disease is well-established, the evidence linking these factors to hypertension is limited [17, 18]. While recent studies have explored the association between reproductive factors and cardiometabolic health [19, 20], critical knowledge gaps remain. Firstly, prior research has often focused on individual cardiometabolic diseases or isolated reproductive events, lacking a systematic investigation of CMM in relation to a comprehensive set of reproductive factors. Secondly, the roles of key exposures such as reproductive span length and specific types of pregnancy loss remain inadequately defined. Thirdly, for studies utilizing the same large cohort, extended follow-up time, more refined definitions of exposures and outcomes, and deeper analytical approaches can provide updated and more robust evidence. Building on this, we propose an integrative life-course hypothesis: that reproductive characteristics indicative of a shortened reproductive lifespan and increased birth load may systematically elevate CMM risk [21–23]. Specifically, we postulate that factors related to the timing of menarche and menopause primarily influence CMM risk through hormonal pathways, including altered patterns of endogenous estrogen exposure [24, 25]. Pregnancy-related factors such as parity and younger maternal age may operate via socio-behavioral pathways, reflecting differences in socioeconomic status and health-related behaviors [26]. Adverse pregnancy outcomes may be linked to inflammatory or immunological pathways [27]. These hypothesized pathways are not mutually exclusive and may cumulatively contribute to CMM risk (Supplementary Fig. 1).

Understanding the correlation between woman-specific risk factors and CMM risk can improve our knowledge of CMM pathogenesis and contribute to the development of early-stage risk modification strategies. Therefore, this study aims to examine the associations between a comprehensive set of female reproductive factors and the risk of new-onset CMM, as well as each cardiometabolic disease, using a large cohort of women from the UK Biobank study.

## Methods

### Study population

The UK Biobank is an extensive and detailed prospective study comprising over 500,000 participants aged 40–69 years at recruitment between 2006 and 2010. Baseline assessments were conducted for all participants across 22 assessment centers and included a computer-assisted interview using a touch-screen questionnaire, a physical examination, and the collection of biological samples. The UK Biobank received ethics approval from the North West Multi-Centre Research Ethics Committee, the National Information Governance Board for Health and Social Care in England and Wales, and the Community Health Index Advisory Group in Scotland [28]. All participants provided written informed consent before enrollment, and those who later withdrew from the study were excluded from the present cohort analysis.

Among the women in the UK Biobank (*N* = 273,326), we excluded those who withdrew from the study (*N* = 32), those with a history of any of the cardiometabolic conditions under study–ischemic heart disease, type 2 diabetes, stroke, or hypertension (*N* = 24,162), and those lacking information on reproductive factors (*N* = 509). Finally, 248,623 women were included in the analysis (Supplementary Fig. 2). The number of participants varied for each analysis of specific sex-related risk factors due to missing values for certain risk factors.

### Assessment of female sex-specific risk factors

Self-reported reproductive factors examined in this study included age at menarche, age at menopause, history of irregular menstrual cycle (yes or no), total reproductive years, years after menopause, maternal age at the first and last live birth, number of live births, history of stillbirth, spontaneous miscarriage or termination (yes or no), number of stillbirths, number of spontaneous miscarriages. Total reproductive years were defined as the duration between menopause age and menarche age. Years after menopause was defined as the duration between the menopause age and the baseline age. Responses such as “Prefer not to answer” and “Do not know” were categorized as “missing” data. Subsequently, in order to explore the combined and cumulative effects of female sex-specific risk factors, among the population with complete data on exposure variables, the significant risk factors identified in the primary results were further transformed into binary variables (using cut-offs derived from internal risk patterns and aligned with existing literature) including [29–31]: early menarche (age at menarche < 12 years, yes or no), early menopause (age at menopause < 50 years, yes or no), short reproductive years (total reproductive years < 31 years, yes or no), younger live birth age (maternal age at live birth < 26 years, yes or no), many live births (number of live births > 2, yes or no), adverse pregnancy outcomes (have a history of stillbirth, spontaneous miscarriage or termination, yes or no).

### Definition of CMM

In this study, CMM was defined as the presence of at least two of the following four CMDs: T2D, IHD, stroke and hypertension, in accordance with previous studies [6, 32–34]. Data on these conditions were obtained from self-reported, physician-diagnosed diseases and hospital inpatient records, collected by the UK Biobank. Diagnoses were coded using the International Classification of Disease, Ninth and Tenth Revisions (ICD-9 and ICD-10), self-reported information at baseline was used as a supplement to disease diagnoses, as summarized in Supplementary Table 1 [35, 36]. Follow-up continued until October 31, 2022. Participants were tracked until one of the following occurred: the end of follow-up, the onset of CMM, death, or loss to follow-up, whichever occurred first. The date of incident CMM was correspondingly defined as the date of diagnosis of the second qualifying CMD. To account for competing risks, individuals who died from non-cardiometabolic diseases were censored at the time of death.

### Measurement of other covariates

We included several covariates in our analysis based on prior research [29, 30]. These covariates were selected to account for potential confounding factors. The variables considered included age, race (categorized as White population and other races), education level (categorized as degree or above, any other qualification, and no qualification), and socioeconomic status measured using the Townsend Deprivation Index (TDI) were among the variables considered.

Additional covariates included smoking status (categorized as never smoking, former smoking, and current smoking), alcohol use status (categorized as never, former, and current drinkers), body mass index (BMI, calculated as weight [kg]/height [m]^2^) and lipid concentrations (high-density lipoprotein cholesterol (HDL cholesterol), low-density lipoprotein cholesterol (LDL cholesterol), measured using the Beckman Coulter AU580). Use of cholesterol-lowering medication, hormone therapy, and contraceptive medication was considered as covariates. Further details on data collection methods are available elsewhere [28].

### Statistical analysis

For baseline participant characteristics, continuous variables were summarized using means and standard deviation (SD), while categorical variables were presented as frequencies and percentages.

We employed multivariable Cox proportional hazards regression models to assess the associations between each risk factor and incident CMM and each cardiometabolic disease. Model 1 adjusted for baseline age, while model 2 additionally adjusted for race, education level, TDI, smoking status, alcohol use status, BMI, HDL cholesterol, LDL cholesterol, use of cholesterol-lowering medication, use of hormone therapy, and use of contraceptive medication.

Multiple adjusted restricted cubic splines (using 4 knots) were constructed to explore the potential nonlinearity in the associations of sex-specific risk factors (analyzed on a continuous scale) and incident CMM, accompanied by effect plots to visualize nonlinear relationships. Based on the shape of these dose–response curves and informed by thresholds commonly used in prior literature [29, 30], cutoff values were determined for each risk factor to create categorical variables. These categorical variables were then utilized in subsequent Cox proportional hazards regression models to quantify the nonlinear associations.

We defined six binary "high-risk reproductive profiles" were defined based on the primary analysis. An UpSet plot was then utilized to visualize the distribution of all observed combinations of these profiles within the cohort. Subsequently, Cox proportional hazards regression model was employed to assess the relationships between the combinations and accumulations of different risk factors and the occurrence of CMM.

Furthermore, subgroup analyses were conducted stratified by baseline age, Townsend deprivation index and BMI. These analyses aimed to explore potential effect modification and enhance understanding of the relationships between risk factors and incident CMM within specific demographic and socioeconomic strata.

In sensitivity analyses, we repeated all of the analyses among participants without missing of covariate data at baseline to verify the robustness of our main result. Then, recognizing that hysterectomy or oophorectomy may influence birth-related characteristics, menopause, and result in significant variations in sex hormone levels, we performed sensitivity analyses by excluding women who had undergone these procedures. Meanwhile, to minimize the likelihood of reverse causality, we also conducted sensitivity analyses excluding female participants within the first 2 years of follow-up. Next, to investigate the role of sex hormones in the linear association between sex-specific risk factors and CMM, model 2 was adjusted for serum concentrations of testosterone and sex hormone–binding globulin. Then, considering that the restriction of the sample to participants without any CMD conditions at baseline may be introducing bias or leading to an underestimation of the effects, we included participants with one type of CMD at baseline in the analysis. To clarify the independent effect of maternal age at birth, we further adjusted for number of live births and reassessed the associations of age at first and last live birth with CMM risk. Finally, to assess the robustness of the chosen cutoffs, we conducted sensitivity analyses using alternative clinically relevant thresholds informed by previous studies.

Missing values in confounding variables were imputed using the multiple interpolation imputation method via the “mice” package in R. Detailed missing rates for the exposure variables and covariates used in this study are provided in Supplementary Table 2–3. Two-sided *P* values were considered significant at *P* < 0.05. All statistical analyses were performed using R version 4.2.2.

## Results

### Baseline characteristics

A total of 248,623 women without CMM at baseline were included in the study, with a mean (SD) age of 56.4 (8.0) years. The baseline characteristics of the study population are presented in Table 1. The median (IQR) follow-up period was 13.6 (12.8–14.4) years, during which 15,807 (6.36%) women developed CMM. Participants with CMM were found to be older, have lower educational attainment, and possess lower socioeconomic status compared to those without CMM. Regarding sex-specific risk factors, people with CMM had earlier age at menarche and menopause, shorter total reproductive years, earlier age at first and last live birth, as well as longer years after menopause compared to non-CMM populations. The mean (SD) age of the population with complete female sex-specific risk factor variables was 60.8 (5.3) years, of which 7,305(7.72%) women developed CMM, and the population characteristics were roughly consistent with the whole population, as shown in Supplementary Table 4.

**Table 1:** Baseline characteristics of the study population

	**Total (*N* = 248,623)**	**No CMM(*N* = 232,816)**	**CMM(*N* = 15,807)**	***P* value**
Age, mean (SD)	56.4 (8.0)	56.1 (8.0)	61.0 (6.8)	< 0.001
Race (%)				< 0.001
Non-white	12,767 (5.1%)	11,441 (4.9%)	1326 (8.4%)	
White	235,182 (94.9%)	220,753 (95.1%)	14,429 (91.6%)	
Education level (%)				< 0.001
No qualification	38,666 (15.8%)	34,100 (14.9%)	4566 (29.8%)	
Any other qualification	125,792 (51.5%)	118,185 (51.6%)	7607 (49.6%)	
Degree or above	79,863 (32.7%)	76,708 (33.5%)	3155 (20.6%)	
Townsend deprivation index, mean (SD)	−1.4 (3.0)	−1.4 (3.0)	−0.8 (3.3)	< 0.001
Smoking status (%)				< 0.001
No smoking history	148,781 (60.1%)	140,298 (60.5%)	8483 (54.0%)	
Past smoking history	76,822 (31.0%)	71,482 (30.8%)	5340 (34.0%)	
Current smoking history	22,145 (8.9%)	20,248 (8.7%)	1897 (12.1%)	
Alcohol use status (%)				< 0.001
Never	21,936 (8.8%)	19,490 (8.4%)	2446 (15.5%)	
Former	133,013 (53.5%)	123,879 (53.2%)	9134 (57.9%)	
Current	93,480 (37.6%)	89,275 (38.4%)	4205 (26.6%)	
BMI (%)				< 0.001
BMI < 25	101,734 (41.3%)	98,570 (42.7%)	3164 (20.2%)	
25 ≤ BMI < 30	91,258 (37.0%)	85,618 (37.1%)	5640 (36.0%)	
BMI ≥ 30	53,514 (21.7%)	46,661 (20.2%)	6853 (43.8%)	
HDL cholesterol, mean (SD)	1.6 (0.4)	1.6 (0.4)	1.5 (0.4)	< 0.001
LDL cholesterol, mean (SD)	3.7 (0.9)	3.7 (0.8)	3.6 (1.0)	< 0.001
Use of cholesterol-lowering medication (%)				< 0.001
No	222,845 (90.8%)	212,465 (92.4%)	10,380 (67.0%)	
Yes	22,605 (9.2%)	17,504 (7.6%)	5101 (33.0%)	
Use of hormone therapy (%)				< 0.001
No	156,702 (63.2%)	148,998 (64.2%)	7704 (49.0%)	
Yes	91,054 (36.8%)	83,050 (35.8%)	8004 (51.0%)	
Use of contraceptive medication (%)				< 0.001
No	45,002 (18.2%)	40,704 (17.5%)	4298 (27.3%)	
Yes	202,892 (81.8%)	191,463 (82.5%)	11,429 (72.7%)	
Age at menarche, mean (SD)	13.0 (1.6)	13.0 (1.6)	12.9 (1.7)	< 0.001
History of irregular menstrual cycle				0.004
No	38,067 (80.8%)	37,353 (80.9%)	714 (77.1%)	
Yes	9022 (19.2%)	8810 (19.1%)	212 (22.9%)	
Age at menopause, mean (SD)	49.7 (5.0)	49.8 (5.0)	49.2 (5.8)	< 0.001
Total reproductive years, mean (SD)	36.8 (5.3)	36.8 (5.2)	36.2 (6.1)	< 0.001
Years after menopause	10.6(6.8)	10.4(6.7)	13.5(7.3)	< 0.001
Age at first live birth, mean (SD)	25.5 (4.6)	25.6 (4.6)	23.9 (4.3)	< 0.001
Age at last live birth, mean (SD)	30.4 (4.9)	30.5 (4.9)	29.4 (4.9)	< 0.001
No. of live births (%)				< 0.001
0–1	80,955 (32.6%)	76,609 (32.9%)	4346 (27.6%)	
2	108,950 (43.9%)	102,383 (44.0%)	6567 (41.6%)	
3	43,418 (17.5%)	40,181 (17.3%)	3237 (20.5%)	
≥ 4	15,023 (6.0%)	13,402 (5.8%)	1621 (10.3%)	
History of stillbirth, spontaneous miscarriage or termination				0.004
No	165,218 (67.6%)	154,905 (67.6%)	10,313 (66.5%)	
Yes	79,279 (32.4%)	74,090 (32.4%)	5189 (33.5%)	
No. of stillbirth (%)				< 0.001
0	73,048 (92.4%)	68,497 (92.7%)	4551 (88.2%)	
1	5214 (6.6%)	4701 (6.4%)	513 (9.9%)	
≥ 2	783 (1.0%)	685 (0.9%)	98 (1.9%)	
No. of spontaneous abortion (%)				< 0.001
0	28,348 (35.9%)	26,681 (36.2%)	1667 (32.4%)	
1	36,369 (46.1%)	33,995 (46.1%)	2374 (46.1%)	
≥ 2	14,173 (18.0%)	13,068 (17.7%)	1105 (21.5%)	

*Abbreviation*: *CMM* cardiometabolic multimorbidity, *BMI* body mass index, *HDL* cholesterol, high density lipoprotein cholesterol, *LDL* cholesterol, low density lipoprotein cholesterol, *SD* standard deviation

### Association of female sex-specific risk factors with CMM and four cardiometabolic diseases

#### Factors related to menarche and menopause

**Fig. 1:**
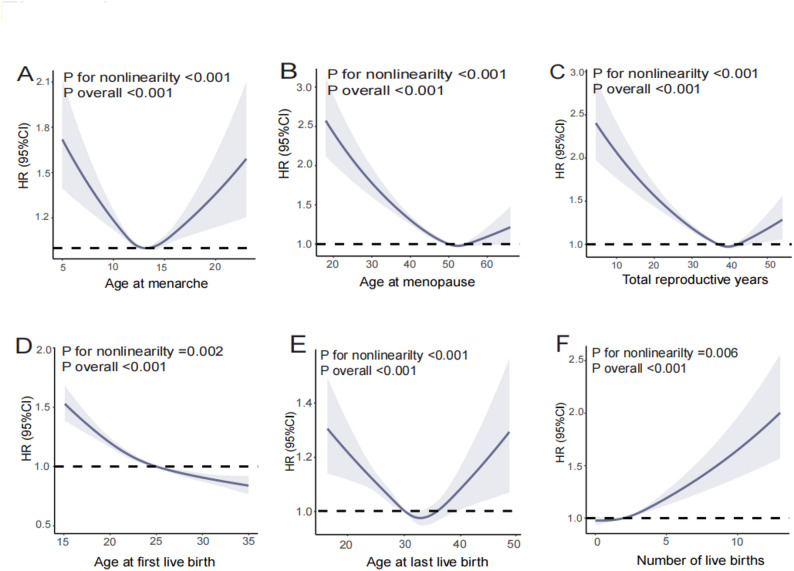
Effect plots for nonlinear associations of female sex-specific risk factors with cardiometabolic multimorbidity. **A** Age at menarche; **B** Age at menopause; **C** Total reproductive years; **D** Age at first live birth; **E** Age at last live birth; **F** Number of live births. Model was adjusted for baseline age, race, educational level, Townsend deprivation index, body mass index, high-density lipoprotein cholesterol, low-density lipoprotein cholesterol, smoking status, alcohol use status, use of cholesterol-lowering medication, use of hormone therapy, and use of contraceptive medication. Blue shaded areas indicate 95% CIs. HR indicates hazard ratio.Abbreviation: HR, hazard ratio; CI, confidence interval

Age at menarche, age at menopause, and total reproductive years showed nonlinear associations with incident CMM **(**Fig. 1A-C). Factors related to menarche and menopause both exhibited U-shaped relationships with the risk of CMM, implying that both excessively low and high levels of exposure are linked to increased risk (*P* for nonlinearity < 0.001). Early menarche, particularly before the age of 12 years (HR, 1.11; 95% CI, 1.06–1.15, *P* < 0.001), was associated with a higher risk of developing CMM compared with menarche occurring between the ages of 12 and 13 years. Similarly, an early menopause (age < 35 years: HR, 1.80; 95% CI, 1.58–2.06, *P* < 0.001; age 35–44 years: HR, 1.24; 95% CI, 1.17–1.32, *P* < 0.001; age 45–49 years: HR, 1.09; 95% CI, 1.04–1.15, *P* < 0.001) was associated with increased CMM risk. A shorter duration of total reproductive year, defined as 20 years or less (HR, 1.78; 95% CI, 1.53–2.08, *P* < 0.001) and 21 to 30 years (HR, 1.22; 95% CI, 1.15–1.29, *P* < 0.001), was markedly associated with higher CMM risks. Additionally, more years since menopause were associated with a higher risk of CMM (HR, 1.02; 95% CI, 1.01–1.02, *P* < 0.001) (Table 2).

**Table 2:** Association between female sex-specific risk factors and risk of cardiometabolic multimorbidity

Characteristic	Total	Case/Person-Years	Model 1		Model 2	
			**HR (95% CI)**	***P* value**	**HR (95% CI)**	***P* value**
Factors Related to Menarche and Menopause						
Age at menarche, years						
< 12	47,133	3591/612908	1.28(1.23–1.34)	< 0.001	1.11(1.06–1.15)	< 0.001
12–13	105,420	6177/1386175	1.00 (Ref.)	-	1.00 (Ref.)	-
> 13	88,584	5521/1162166	1.04(1.00–1.07)	0.059	1.02(0.98–1.06)	0.289
Irregular menstrual cycle vs not	9022	212/122106	1.25(1.07–1.46)	0.005	1.10(0.94–1.28)	0.229
Age at menopause, years						
< 35	1609	236/19975	2.61(2.29–2.97)	< 0.001	1.80(1.58–2.06)	< 0.001
35–44	16,020	1537/205090	1.53(1.45–1.63)	< 0.001	1.24(1.17–1.32)	< 0.001
45–49	33,574	2467/435781	1.19(1.13–1.25)	< 0.001	1.09(1.04–1.15)	< 0.001
50–54	67,175	4493/875931	1.00 (Ref.)	-	1.00 (Ref.)	-
55–59	19,763	1591/255014	1.05(0.99–1.12)	0.076	1.02(0.96–1.08)	0.507
≥ 60	841	93/10348	1.19(0.97–1.46)	0.096	1.06(0.87–1.31)	0.549
Total reproductive years, years						
≤ 20	1100	166/13656	2.56(2.19–2.99)	< 0.001	1.78(1.53–2.08)	< 0.001
21–30	14,511	1413/185621	1.45(1.37–1.54)	< 0.001	1.22(1.15–1.29)	< 0.001
31–40	89,340	6183/1162872	1.00 (Ref.)	-	1.00 (Ref.)	-
> 40	30,721	2394/397372	1.01(0.96–1.05)	0.833	0.98(0.94–1.03)	0.498
Years after menopause, years	248,623	15,807/3258598	1.03(1.03–1.04)	< 0.001	1.02(1.01–1.02)	< 0.001
Factors Related to Pregnancy and Childbirth						
Age at first live birth, years						
< 21	24,252	2631/309710	2.15(2.05–2.26)	< 0.001	1.35(1.28–1.42)	< 0.001
21–25	62,864	5007/818386	1.41(1.35–1.47)	< 0.001	1.13(1.08–1.18)	< 0.001
> 25	79,943	3742/1059636	1.00 (Ref.)	-	1.00 (Ref.)	-
Age at last live birth, years						
< 26	27,329	2555/352225	1.38(1.31–1.46)	< 0.001	1.12(1.06–1.18)	< 0.001
26–28	34,121	2639/444767	1.11(1.05–1.17)	< 0.001	1.03(0.97–1.08)	0.332
29–31	40,039	2596/525913	1.00 (Ref.)	-	1.00 (Ref.)	-
32–35	38,923	2110/513895	0.94(0.88–0.99)	0.024	0.96(0.91–1.02)	0.179
> 35	26,374	1425/347620	1.03(0.96–1.09)	0.437	1.04(0.97–1.11)	0.275
Number of live births						
0–1	80,955	4346/1063275	1.00 (Ref.)	-	1.00 (Ref.)	-
2	108,950	6567/1432869	0.93(0.9–0.97)	< 0.001	0.98(0.94–1.02)	0.343
3	43,418	3237/567193	1.10(1.05–1.15)	< 0.001	1.05(1.00–1.10)	0.044
≥ 4	15,023	1621/191774	1.55(1.47–1.64)	< 0.001	1.19(1.13–1.27)	< 0.001
Stillbirth, spontaneous miscarriage or termination vs not	79,279	5189/1036477	1.16(1.12–1.19)	< 0.001	1.12(1.08–1.15)	< 0.001
Number of stillbirths						
0	73,048	4551/957129	1.00 (Ref.)	-	1.00 (Ref.)	-
1	5214	513/66673	1.28(1.17–1.40)	< 0.001	1.07(0.97–1.17)	0.172
≥ 2	783	98/9693	1.85(1.51–2.25)	< 0.001	1.30(1.07–1.59)	0.010
Number of spontaneous miscarriages						
0	28,348	1667/370614	1.00 (Ref.)	-	1.00 (Ref.)	-
1	36,369	2374/476880	0.99(0.93–1.05)	0.662	1.04(0.97–1.10)	0.263
≥ 2	14,173	1105/184048	1.24(1.15–1.34)	< 0.001	1.24(1.15–1.34)	< 0.001

Model 1 was adjusted for baseline age

Model 2 was adjusted for baseline age, race, educational level, Townsend deprivation index, body mass index, high-density lipoprotein cholesterol, low-density lipoprotein cholesterol, smoking status, alcohol use status, use of cholesterol-lowering medication, use of hormone therapy and use of contraceptive medication

*Abbreviation*: *HR* hazard ratio, *CI* confidence interval,*Ref* reference

Women who experienced menarche before the age of 12 years were at a higher risk of developing IHD, T2D and hypertension (IHD: HR, 1.11; 95% CI, 1.06–2.16, *P* < 0.001; T2D: HR, 1.12; 95% CI, 1.06–1.18, *P* < 0.001; hypertension: HR, 1.10; 95% CI, 1.07–1.12, *P* < 0.001) compared to those who had menarche at a later age. Similarly, an early age at menopause was associated with a greater risk of all four cardiometabolic diseases (IHD,T2D,hypertension and stroke). A shorter duration of total reproductive years, defined as 20 years or less and 21 to 30 years, was markedly associated with higher four cardiometabolic diseases risks. In addition, we found that total reproductive years greater than 40 years were associated with an increased risk of developing hypertension. The longer years after menopause were associated with a higher risk of four cardiometabolic diseases (Supplementary Table 5–6).

#### Factors related to pregnancy and childbirth

Maternal age at first and last live births, as well as the number of live births, showed nonlinear associations with incident CMM **(**Fig. 1D-F**)**. A U-shaped association was identified between maternal age at last live births and the risk of new-onset CMM (*P* for nonlinearity < 0.001). A younger maternal age at first live birth (age < 21 years: HR, 1.35; 95% CI, 1.28–1.42, *P* < 0.001; age 21–25 years: HR, 1.13; 95% CI, 1.08–1.18, *P* < 0.001) and a younger age at last live birth (age < 26 years: HR, 1.12; 95% CI, 1.06–1.18, *P* < 0.001) were associated with an elevated risk of CMM. Figure 1F illustrates a J-shaped association between number of live births and incident CMM (*P* for nonlinearity < 0.001). Compared with women who had 0 or 1 live births, the risk of CMM was higher among those with 3 live births (HR, 1.05; 95% CI, 1.00–1.10, *P* = 0.044) and significantly higher among women with 4 or more live births (HR, 1.19; 95% CI, 1.13–1.27, *P* < 0.001). Women who experienced stillbirth, spontaneous miscarriage or pregnancy termination of pregnancy (HR, 1.12; 95% CI, 1.08–1.15, *P* < 0.001) had an increased risk of developing CMM. Furthermore, women who experienced more than 2 stillbirths had an increased risk of developing CMM compared to those without stillbirth (HR, 1.30; 95% CI, 1.07–1.59, *P* = 0.010), and those with 2 or more spontaneous miscarriages faced a 1.24-fold increase in CMM risk (95% CI: 1.15–1.34, *P* < 0.001) compared to women without a history of miscarriage (Table 2).

A younger maternal age at first and last live birth were associated with an increased risk of the four cardiometabolic diseases. Compared with women with 0 or 1 live birth, the risk of IHD and hypertension was higher among those with three live births (IHD:HR, 1.09; 95% CI, 1.03–1.15, *P* = 0.002; hypertension: HR, 1.04; 95% CI, 1.02–1.07, *P* = 0.001). Women with four or more live births had an elevated risk for all four cardiometabolic diseases. Additionally, women who experienced stillbirth, spontaneous miscarriage, or pregnancy termination had a higher risk of developing IHD, T2D and hypertension (IHD: HR, 1.12; 95% CI, 1.08–1.16, *P* < 0.001; T2D: HR, 1.08; 95% CI, 1.03–1.12, *P* < 0.001; hypertension: HR, 1.08; 95% CI, 1.06–1.10, *P* < 0.001). Furthermore, the risk of hypertension was increased in women who had one or more stillbirth compared to those without a history of stillbirth. Women with two or more spontaneous miscarriages faced an increased risk of all four cardiometabolic diseases compared to those without a history of miscarriage (Supplementary Table 5–6).

### Association of combinations and accumulations of female sex-specific risk factors with CMM

#### Factors related to menarche and menopause

**Fig. 2:**
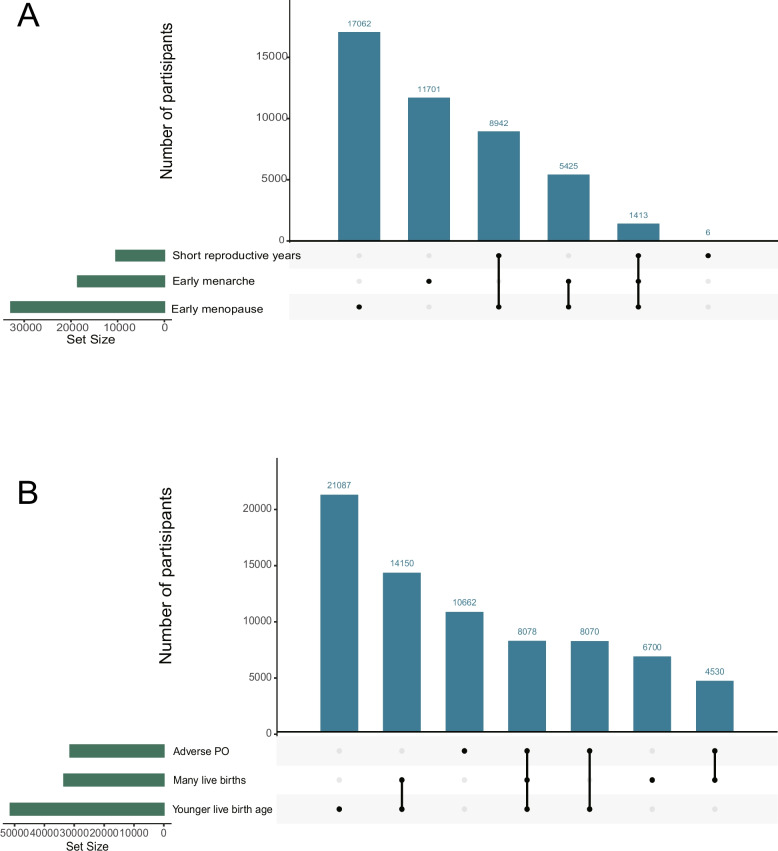
Combination patterns of female sex-specific risk factors. **A** Combination patterns of factors related to menarche and menopause. **B** Combination patterns of factors related to pregnancy and childbirth. Early menarche (age at menarche < 12 years, yes or no), Early menopause (age at menopause < 50 years, yes or no), Short reproductive years (total reproductive years < 31 years, yes or no), Younger live birth age (maternal age at live birth < 26 years, yes or no), Many live births (number of live births > 2, yes or no), Adverse PO (have a history of stillbirth, spontaneous miscarriage or termination, yes or no). Abbreviation: PO, pregnancy outcomes

Early menarche accompanied by short reproductive years (HR, 1.26; 95% CI, 1.17–1.36, *P* < 0.001), early menarche accompanied by early menopause (HR, 1.22; 95% CI, 1.11–1.34, *P* < 0.001), and the simultaneous presence of early menarche, early menopause, and short reproductive years (HR, 1.66; 95% CI, 1.44–1.91, *P* < 0.001) are three combination patterns of risk factors related to menarche and menopause **(**Fig. 2), and are associated with the occurrence of CMM (Table 3). Additionally, as the number of female risk factors increases, the risk of developing CMM also gradually rises (*P* for trend < 0.001) (Table 4).

**Table 3:** Association between different combinations of female sex-specific risk factors and risk of cardiometabolic multimorbidity

Characteristic	Total	Case/Person-Years	Model 1		Model 2	
			**HR (95% CI)**	***P* value**	**HR (95% CI)**	***P* value**
Factors Related to Menarche and Menopause						
None	50,027	3465/652555	1.00 (Ref.)	-	1.00 (Ref.)	-
Early menarche + Early menopause	5425	513/69743	1.54(1.41–1.69)	< 0.001	1.22(1.11–1.34)	< 0.001
Early menopause + Short reproductive years	8942	897/114567	1.56(1.45–1.68)	< 0.001	1.26(1.17–1.36)	< 0.001
Early menarche + Early menopause + short reproductive years	1413	210/17615	2.40(2.09–2.76)	< 0.001	1.66(1.44–1.91)	< 0.001
Factors Related to Pregnancy and Childbirth						
None	21,299	1184/279867	1.00 (Ref.)	-	1.00 (Ref.)	-
Yonger live birth age + Many live birth	14,150	1483/181009	1.70(1.57–1.83)	< 0.001	1.22(1.13–1.32)	< 0.001
Yonger live birth age + Adverse PO	8070	723/103601	1.62(1.47–1.77)	< 0.001	1.26(1.15–1.38)	< 0.001
Many live birth + Adverse PO	4530	279/59078	1.16(1.02–1.33)	0.023	1.15(1.01–1.31)	0.037
Yonger live birth age + Many live birth + adverse PO	8078	913/102544	1.96(1.80–2.14)	< 0.001	1.35(1.23–1.47)	< 0.001

None, no female sex-specific risk factors

Model 1 was adjusted for baseline age

Model 2 was adjusted for baseline age, race, educational level, Townsend deprivation index, body mass index, high-density lipoprotein cholesterol, low-density lipoprotein cholesterol, smoking status, alcohol use status, use of cholesterol-lowering medication, use of hormone therapy, and use of contraceptive medication

*Abbreviation*: *PO* pregnancy outcomes, *HR* hazard ratio, *CI* confidence interval; Ref., reference

**Table 4:** Association between female sex-specific risk factors accumulation and risk of cardiometabolic multimorbidity

Characteristic	Total	Case/Person-Years	Model 1		Model 2	
			**HR (95% CI)**	***P* value**	**HR (95% CI)**	***P* value**
Factors Related to Menarche and Menopause						
0	50,027	3465/652555	1.00 (Ref.)	-	1.00 (Ref.)	-
1	28,769	2220/373026	1.19(1.13–1.26)	< 0.001	1.09(1.04–1.15)	< 0.001
2	14,367	1410/184310	1.55(1.46–1.65)	< 0.001	1.25(1.17–1.33)	< 0.001
3	1413	210/17615	2.40(2.09–2.76)	< 0.001	1.66(1.44–1.91)	< 0.001
*P* for trend			< 0.001		< 0.001	
Factors Related to Pregnancy and Childbirth						
0	21,299	1184/279867	1.00 (Ref.)	-	1.00 (Ref.)	-
1	38,449	2723/501407	1.24(1.16–1.33)	< 0.001	1.10(1.03–1.18)	< 0.001
2	26,750	2485/343688	1.59(1.48–1.70)	< 0.001	1.22(1.14–1.31)	< 0.001
3	8078	913/102544	1.96(1.80–2.14)	< 0.001	1.35(1.23–1.47)	< 0.001
*P* for trend			< 0.001		< 0.001	

Model 1 was adjusted for baseline age

Model 2 was adjusted for baseline age, race, educational level, Townsend deprivation index, body mass index, high-density lipoprotein cholesterol, low-density lipoprotein cholesterol, smoking status, alcohol use status, use of cholesterol-lowering medication, use of hormone therapy, and use of contraceptive medication

*Abbreviation*: *HR* hazard ratio, *CI* confidence interval, *Ref*, reference

#### Factors related to pregnancy and childbirth

Younger live birth age accompanied by many live births(HR, 1.22; 95% CI, 1.13–1.32, *P* < 0.001), younger live birth age accompanied by adverse pregnancy outcomes(HR, 1.26; 95% CI, 1.15–1.38, *P* < 0.001), many live births accompanied by adverse pregnancy outcomes(HR, 1.15; 95% CI, 1.01–1.31, *P* = 0.037), and the simultaneous occurrence of younger live birth age, many live births and adverse pregnancy outcomes(HR, 1.35; 95% CI, 1.23–1.47, *P* < 0.001) are four combination patterns of risk factors related to childbirth and pregnancy (Fig. 2), and are associated with the occurrence of CMM (Table 3). Similarly, as the number female of risk factors increases, the risk of developing CMM also rises gradually (*P* for trend < 0.001) (Table 4).

### Stratified analysis results

We conducted stratified analyses based on baseline age, socioeconomic status (SES) and obesity status to explore the associations of sex-specific risk factors with CMM. The results indicated that these associations were more likely to produce significant HRs in subgroups characterized by a baseline age of 60 years or older, lower SES, and a BMI of 30 kg/m^2^ or higher.

Age-stratified analyses suggested significant interactions between age at menarche and baseline age (*P* for interaction < 0.001). Meanwhile significant interactions were also observed between baseline age and several reproductive factors, including age at first live birth, age at last live birth, number of live births and number of stillbirths (*P* for interaction < 0.001, 0.004, < 0.001, and 0.038, respectively). When stratifying by the Townsend deprivation index, an interaction was identified between SES and years after menopause (higher SES: HR, 1.02; 95% CI, 1.01–1.02; lower SES: HR, 1.02; 95% CI, 1.01–1.02; *P* for interaction < 0.001). In BMI-stratified analyses, interactions were observed between BMI and several reproductive factors, including age at menarche, years after menopause, age at first live birth, age at last live birth and number of live births (*P* for interactio*N* = 0.033, < 0.001, 0.003, < 0.001, and 0.004, respectively). (Supplementary Table 7–9).

### Sensitivity analyses

Several sensitivity analyses were performed to assess the robustness of the main results. The complete case analysis generally exhibited trends consistent with those obtained from multiple imputation. However, no association was found between 3 live births and CMM (Supplementary Table 10). To further mitigate the potential impact of reverse causality, additional sensitivity analyses were carried out by excluding female participants within the initial 2 years of follow-up, yielding results consistent with the primary findings. For example, the age at menarche < 12 years was significantly associated with higher CMM risk (HR, 1.10; 95% CI, 1.05–1.15). **(**Supplementary Table 11). The influence of hysterectomy or oophorectomy on artificial menopause was investigated by excluding individuals who had undergone these procedures. Compared with the initial results, the association between stillbirth and the incident CMM became nonsignificant, while the effect sizes of the other reproductive factors remained largely unchanged. **(**Supplementary Table 12**)**. Additionally, further adjustment for blood sex hormones levels did not substantially alter the associations between each risk factor and CMM. Concurrently, early menopause demonstrated a stronger effect size in relation to CMM risk (age < 35 years: HR, 1.82; 95% CI, 1.60–2.08; age 35–44 years: HR, 1.25; 95% CI, 1.18–1.32). **(**Supplementary Table 13). In the analysis that included participants with one type of CMD at baseline, we found that the effect size of reproductive factors increased moderately (Supplementary Table 14). After further adjustment for number of live births, the direction, statistical significance, and magnitude of the associations between age at first live birth (age < 21 years: HR, 1.31; 95% CI, 1.24–1.39; age 21–25 years: HR, 1.12; 95% CI, 1.07–1.17) and age at last live birth (age < 26 years: HR, 1.15; 95% CI, 1.08–1.21) with CMM risk remained materially unchanged **(**Supplementary Table 15). After evaluating alternative cutoffs, we found that the main conclusions remained materially unchanged(Supplementary Table 16).

## Discussion

In this large population-based cohort study, we observed that female reproductive history is not only a risk indicator for a single disease, but also an important and comprehensive risk indicator for CMM. Specifically, earlier age at menarche and menopause, shorter total reproductive years, longer years after menopause, younger maternal age at first or last live birth, larger number of live births, and a history of stillbirth, spontaneous miscarriage, or termination were found to be correlated with higher CMM risk. Broadly similar results were found in four cardiometabolic diseases. Meanwhile, our research offers a unique perspective, focusing on the cumulative burden of multiple modifiable reproductive factors. Unlike analyses that only target a single exposure factor or genetic interaction, our systematic approach quantifies the dose–response relationship between the number of adverse reproductive traits and the risk of cardiovascular and metabolic diseases (Supplementary Table 17). This provides a practical phenotypic risk assessment tool that can directly guide clinical stratification.

### Menarche and menopause

Menarche marks the onset of reproductive capacity in women and is influenced by various factors, including genetic, ethnic-racial, nutritional, and living environment [37]. Previous studies have linked earlier menarche to an increased risk of cardiometabolic factors [38] and higher rates of cardiovascular morbidity and mortality [39, 40]. Similarly, the age at menopause has been recognized as a risk factor for cardiovascular mortality [41], with earlier menopause associated with a higher risk of stroke, type 2 diabetes, ischemic heart disease, and hypertension [42–47]. Our findings provide additional evidence linking early menopause to an elevated risk of CMM. This association may be due to reduced lifetime exposure to endogenous estrogens, leading to vascular endothelial dysfunction, inflammatory response, and impaired immune function, which predispose to cardiometabolic disorders [48–50]. Additionally, we found that shorter total reproductive years were associated with a higher risk of CMM and the four cardiometabolic diseases risk, likely due to estrogen deficiency. The nonlinear, approximately U-shaped relationships observed for these factors suggest that deviations from an optimal physiological window in either direction may elevate risk. We also found that both early menarche and a short reproductive lifespan are associated with increased CMM risk, suggesting certain underlying risk factors or conditions associated with a shortened reproductive lifespan may be more common among individuals with early menarche. We propose that these associations primarily operate through a hormonal pathway, whereby altered lifetime estrogen exposure influences vascular function and metabolic health [51].

### Maternal age at live birth

We observed that younger maternal age at either first or last live birth was associated with an increased risk of CMM and the four cardiometabolic diseases, and this association was largely independent of parity. While previous studies have linked younger maternal age at pregnancy with cardiovascular disease [17, 52], such associations with CMM have not yet been established. Furthermore, younger maternal age at first birth tends to be linked to lower education attainment, poorer health status, and a greater burden of comorbidities [53, 54], all of which contribute to an elevated risk of CMM. Previous studies have also reported that early age at first birth was associated with unfavorable cardiovascular risk profiles in both men and women [54, 55]. The association between maternal age at live birth and CMM risk in women may arise not only from biological factors related to pregnancy and childbirth but also from socio-cultural, psychological, and behavioral influences. Therefore, comprehensive interventions targeting the social and behavioral aspects of women who experienced early childbirth are essential to mitigate their CMM risk. We hypothesize that younger maternal age and higher parity influence CMM risk largely through a socio-behavioral pathway, reflecting differences in socioeconomic trajectory and health behaviors [26, 56].

### Number of live births

We observed that a greater number of live births was associated with an increased risk of CMM among parous women. The spline analysis indicated a monotonic increase in CMM risk with rising parity, reinforcing the notion of a cumulative biological or lifestyle burden with each additional pregnancy. Recent pooled study has demonstrated that grand multiparity was a significant risk factor for dementia in women [57], and a meta-analysis indicated that higher parity was significantly associated with an increased risk of type 2 diabetes [58]. These findings suggest that multiparity may have negative long-term effects on woman's health. Pregnancy and childbirth, while natural processes, induce various physiological changes, including alterations in sex hormones, insulin resistance, dyslipidemia, overweight or obesity, and oxidative stress [59–61]. Although these changes are typically temporary and reversible, the long-term hormonal fluctuations and metabolic disturbances associated with multiparity may have latent effects that elevate the risk of future cardiovascular disease [62, 63]. Furthermore, with each pregnancy, the cumulative probability of experiencing specific obstetric complications (such as preeclampsia and gestational diabetes) that are themselves established risk factors for cardiometabolic disease increases, which may further contribute to the long-term risk observed in multiparous women [64, 65].

### Pregnancy complications

In our study, we found that women with a history of stillbirth, spontaneous miscarriage or pregnancy termination faced an elevated risk of developing CMM. Specifically, experiencing two or more stillbirths or spontaneous miscarriages was identified as significant risk factors for CMM. Similarly, stillbirth and spontaneous miscarriage were also risk factors for each cardiometabolic disease.

Pregnancy complications can significantly influence both short-term and long term cardiometabolic trajectories, contributing to adverse cardiovascular outcomes in women [66–69]. A large cohort study using UK Biobank data revealed that a history of miscarriage was associated with a higher risk of coronary heart disease, while a history of stillbirth was linked to an increased risk of cardiovascular disease and stroke [17]. Furthermore, another study demonstrated that pregnancy losses were correlated with subsequent risks of myocardial infarction, cerebral infarction, and renovascular hypertension [70]. These findings highlight the strong association between stillbirth, miscarriage, and cardiovascular disease, suggesting shared underlying vascular pathology characterized by systemic inflammation and endothelial dysfunction. These pathological mechanisms not only contribute to placental dysfunction and pregnancy loss but also play a role in the heightened risk of cardiovascular disease [71, 72]. We propose that adverse pregnancy outcomes increase CMM risk via an inflammatory or immunological pathway, consistent with evidence linking pregnancy loss to chronic inflammation and endothelial dysfunction [27, 73].

### Combination and accumulation of risk factors in women

In our study, we found different combination patterns of multiple risk factors. These may influence CMM through physiological mechanisms such as more severe hormonal imbalances, metabolic disorders, and inflammatory responses [48, 49]. Such as early menarche accompanied by early menopause, may break the normal rhythm of hormones in the female body, so that the cardiovascular system and metabolic function in a long-term unstable state, increasing the risk of CMM [38–41]. The early age of live birth accompanied by many live births means that women bear the pressure of pregnancy and childbirth when their physical development is not fully mature, and the reproductive endocrine system is stimulated several times, resulting in frequent fluctuations in hormone levels, metabolic disorders of the body, and increasing the incidence of CMM [59–61]. At the same time, the accumulation of risk factors will aggravate the imbalance of hormones in the female body, causing a more serious burden on the metabolic system, and the inflammatory response will continue to superposition and continue, further increasing the risk of CMM [62, 63].

### Implications for public health and clinical practice

While our findings align with the established link between reproductive factors and increased cardiometabolic risk [37–45], we observed nuanced variations. The effect sizes for factors like early menopause were somewhat more modest than those reported by Peters et al. [17]. Regarding pregnancy-related factors, our results on younger age at birth support Shan et al. [74] but demonstrate more consistent associations than Yan et al. [75]. These discrepancies are likely attributable to differences in study populations and analytical approaches, including covariate adjustment.

The consistent and graded associations observed in this study, particularly the cumulative risk linked to multiple adverse reproductive factors, have several practical implications. Firstly, in clinical settings, a woman’s reproductive history should be considered a vital sign for her future cardiometabolic health. A simple count of key adverse factors could aid in identifying asymptomatic women who may benefit from earlier and more frequent cardiometabolic monitoring. Secondly, for public health planning, these findings reinforce the need for life-course-centered strategies. This cumulative risk pattern underscores that even individual reproductive factors with modest effect sizes can, in combination, lead to a clinically substantial increase in risk. Therefore, from a public health perspective, these common exposures warrant attention due to their potential aggregate impact at the population level. Finally, our work calls for future research to develop and validate clinical risk scores that incorporate reproductive history and to test the efficacy of targeted interventions in high-risk subgroups defined by these profiles.

### Study strengths and limitations

This study has several strengths, including a large sample size, prospective study design, long follow-up period, comprehensive assessment of sex-specific risk factors for women, and extensive adjustment for various confounding factors. However, several limitations should be considered. First, the study primarily included participants of White European ancestry, which limits the generalizability of our findings to other racial, ethnic, or geographic populations. Reproductive patterns and healthcare contexts vary considerably across settings, and future studies in diverse populations are needed to validate our findings. Second, the restriction of the sample to participants without any CMD conditions at baseline may be introducing bias or leading to an underestimation of the effects. Simultaneously, sex-specific risk factors are likely subject to non-random missingness, and applying a complete-case approach to these variables may introduce systematic selection bias. Additionally, self-reported reproductive histories are subject to recall bias. Because prospective data collection makes any misclassification likely non-differential, the true associations may be stronger than reported. Future validation studies are needed. Third, ICD-based definitions exhibit varying sensitivity and specificity across different diseases, which may lead to different misclassification biases in the definition of outcome CMM. Fourth, although we adjusted for traditional cardiovascular risk factors at baseline, the potential influence of changes in these factors over time cannot be completely ruled out. Fifth, data on certain pregnancy-related factors known to affect the risk of cardiovascular disease later in life, such as breastfeeding, gestational diabetes, and preeclampsia, were not available [66]. Meanwhile, due to the limitations of the data, we were unable to assess some confounding factors, including genetic susceptibility, comorbid conditions such as inflammation or autoimmune diseases and neoplasms, as well as iatrogenic factors. Finally, key reproductive exposures such as age at menopause are inherently time-dependent. Treating them as fixed covariates at baseline may introduce bias, and future studies should employ time-dependent covariate or multi-state models to better capture their dynamic nature.

## Conclusions

This large, population-based cohort study identified sex-specific risk factors associated with the development of new-onset CMM in women. Specifically, early menarche or menopause, younger age at first and last live birth, and having a greater number of live births were associated with an increased risk of CMM. Additionally, both stillbirths and spontaneous miscarriage were associated with a higher risk of incident CMM. Similar associations were observed for each of the four cardiometabolic diseases. At the same time, the combinations and accumulations of different risk factors will further increase the risk of CMM. These findings emphasize the importance of incorporating women's reproductive history into targeted screening strategies for the prevention of cardiometabolic diseases in women.

## Data Availability

The datasets used and/or analysed during the current study are available from the corresponding author on reasonable request.

## Abbreviations

PO: Pregnancy outcomes
BMI: Body Mass Index
CMM: Cardiometabolic Multimorbidity
CMD: Cardiometabolic Disease
CI: Confidence Interval
HDL cholesterol: High-density Lipoprotein Cholesterol
HR: Hazard Ratio
IHD: Ischemic Heart Disease
ICD-9: International Classification of Disease, Ninth
ICD-10: International Classification of Disease, Tenth
IQR: Interquartile Range
LDL cholesterol: Low-density Lipoprotein Cholesterol
PO: Pregnancy Outcomes
SD: Standard Deviation
SES: Socioeconomic Status
T2D: Type 2 Diabetes
TDI: Townsend Deprivation Index
UK Biobank: United Kingdom Biobank
